# Localized Leg Erythema as the Primary Symptom of Scarlet Fever: An Atypical Presentation

**DOI:** 10.7759/cureus.79983

**Published:** 2025-03-03

**Authors:** Yusuke Ito

**Affiliations:** 1 Department of Family Practice, Azusawa Hospital, Itabashi, JPN

**Keywords:** blanchable erythema, group a streptococcus pyogenes, maculopapular exanthema, scarlet fever, streptococcal pharyngitis

## Abstract

Scarlet fever is an acute infectious condition caused by *Streptococcus pyogenes*, commonly leading to dermatological complaints in children. Its classical presentation includes fever, sore throat, and diffuse erythematous macules starting on the trunk; however, rare cases may present primarily with skin manifestations. We report a case of a seven-year-old boy who presented with pruritic, localized leg rashes as the primary symptom of scarlet fever. A thorough physical examination revealed painless erythema of the soft palate. Due to a local outbreak of *Streptococcus pyogenes* infections, a rapid antigen test was performed, yielding a positive result and leading to the diagnosis of scarlet fever. The patient was treated with cefditoren pivoxil, resulting in the complete resolution of symptoms. This case highlights the importance of a thorough patient history, physical examination, and awareness of local epidemiological data in diagnosing scarlet fever.

## Introduction

Skin complaints are among the most common concerns in pediatric care, accounting for nearly 10% of all visits in primary care ambulatory practice [1]. Scarlet fever, an acute infectious disease caused by exotoxin-producing *Streptococcus pyogenes*, is one of the most common dermatologic conditions in children, predominantly affecting those between the ages of three and eight years [1,2]. Population rates of scarlet fever were reported as 24-33 cases per 100,000 in regions such as East Asia and England [3]. Its incidence declined during the COVID-19 pandemic but resurged after mitigation measures [4]. The classical presentation includes diffuse erythematous macules starting on the trunk after the onset of fever and sore throat [5,6]. However, in rare cases, patients may present with atypical rash distributions and report only skin symptoms as the primary complaint. We present an atypical case of scarlet fever with erythema limited to the lower extremities as the primary symptom.

## Case presentation

**Figure 1:**
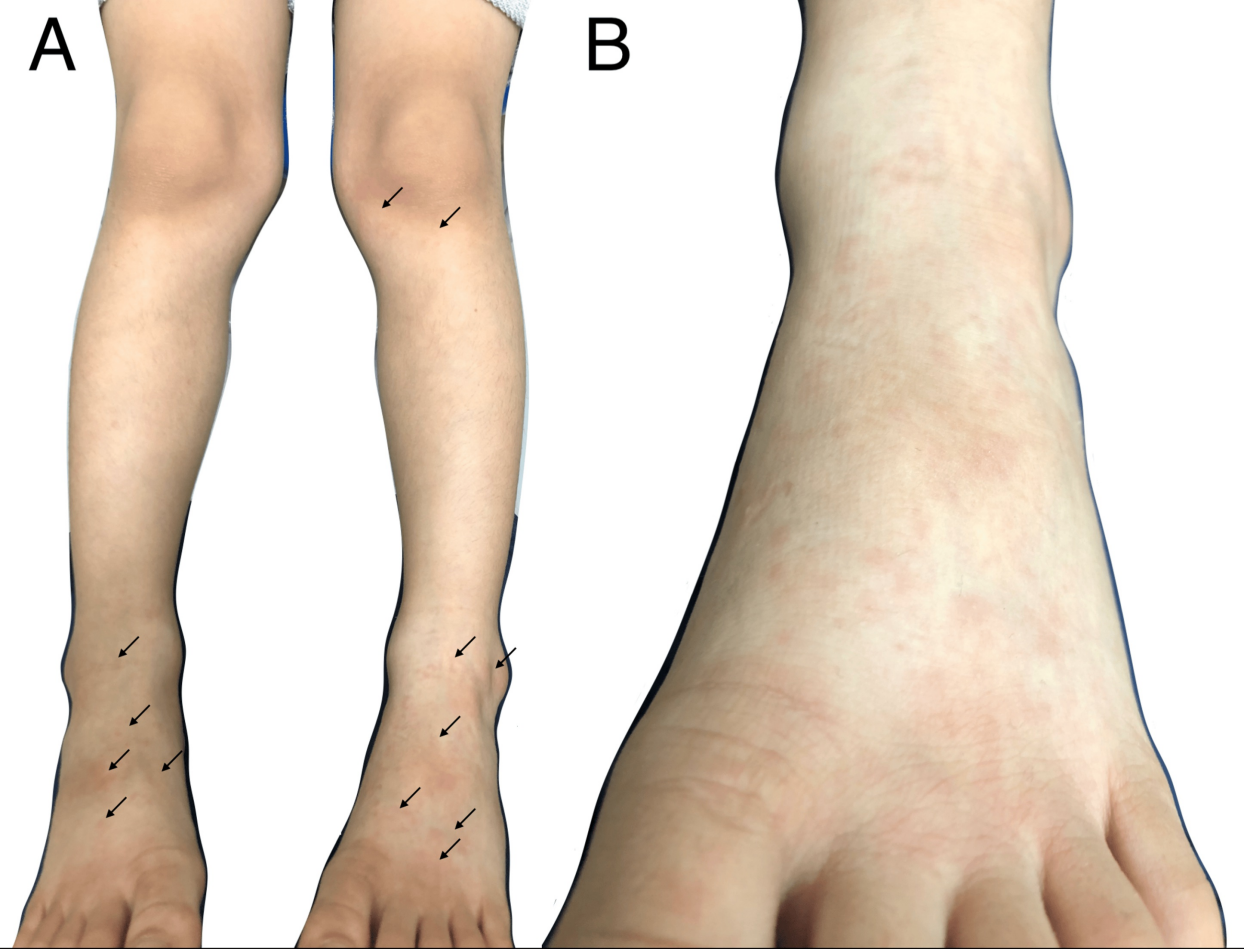
(A) Multiple blanchable erythematous macules were observed on the bilateral dorsal surfaces of the feet and the front of the left lower leg (arrows). (B) A close-up image of the left foot.

A seven-year-old boy presented to our department with leg rashes. On the day of the visit, he complained of mild pruritic rashes on both legs. He reported no prodrome symptoms, including fever and sore throat. Upon presentation, he appeared well and medically stable, with no other symptoms such as sore throat, fever, malaise, or respiratory symptoms. Physical examination revealed no fever on palpation and revealed multiple erythematous macules, measuring 5-10 mm in size, on the front of the left lower leg and bilateral dorsal surfaces of the feet, which blanched on palpation (Figure 1). The soles were spared, with no visible rash in these areas. No other skin abnormalities were observed, including on the face, neck, chest, or upper extremities. A review of systems revealed pharyngeal erythema: painless, pale erythema of the soft palate without petechiae, vesicles, or ulcers. There were no additional findings, such as a red or white strawberry tongue, tonsillitis, or cervical lymphadenopathy.

A consultation with pediatrics indicated that the rash characteristics were nonspecific, and initially, a viral-induced exanthema was considered. However, due to the outbreak of *Streptococcus pyogenes* infections in the surrounding area, it was recommended to rule out streptococcal infection despite the atypical distribution of the rash for scarlet fever. Further history-taking from his parent revealed an outbreak of streptococcal pharyngitis at his daycare center, and the rapid pharyngeal streptococcal antigen test yielded a positive result. There was no history of exposure to other bacterial or viral infections. Based on these findings, and excluding other diseases, a diagnosis of scarlet fever was made. Due to a penicillin shortage, he was treated with cefditoren pivoxil 100 mg twice daily (9 mg/kg/day), leading to an improvement in pruritus and rash. After seven days of treatment, his symptoms resolved completely, and no additional symptoms, including fever, malaise, sore throat, or new rashes, were reported during the treatment period.

## Discussion

Scarlet fever is an acute infectious condition caused by exotoxin-producing strains of group A *Streptococcus* (GAS), also known as *Streptococcus pyogenes* [2]. Hypersensitivity of the patient to exotoxin is involved in the development of scarlet fever [5]. Scarlet fever occurs in approximately 10% of children with streptococcal pharyngitis [5]. Its classical presentation includes fever and sore throat, followed by a rash appearing 12-48 hours later [5]. This rash is characterized by confluent, erythematous, blanching, fine macules, resembling sunburn and sandpaper-like papules [5,6]. The rash typically starts on the chest and abdomen and gradually extends to the extremities [2,6]. In rare cases, skin symptoms or atypical rash distributions may present as the primary complaint; however, to the best of our knowledge, only one adult case has been reported in case reports [7].

The diagnosis of scarlet fever is primarily clinical, based on characteristic findings and evidence of GAS pharyngitis. The Centor score and rapid antigen tests are commonly used for diagnosing GAS pharyngitis, with cultures ordered if there is a high suspicion of GAS pharyngitis and a negative rapid antigen test result [2,5]. Erythema in children can result from various etiologies, including viral, bacterial, and fungal infections, autoimmune diseases, or drug reactions. We highlighted common and clinically significant differential diagnoses (Table 1) [5,8,9]. Diagnosis requires assessing the rash's morphology and distribution, along with medication history, infection exposure, and a thorough physical examination.

**Table 1:** Key differential diagnoses of erythema in children.

Causes	Differentiating features
Measles	During the prodromal phase, fever, the three Cs (cough, coryza, and conjunctivitis), and Koplik spots, followed by a maculopapular erythematous rash starting on the head and neck [8].
Rubella	Generalized maculopapular erythema, resolving within 72 hours, accompanied by retroauricular, posterior cervical, and posterior occipital lymphadenopathy [9].
Roseola infantum	Erythematous maculopapular rash appearing after 3-5 days of high fever, starting on the trunk and spreading peripherally [5,8].
Pityriasis rosea	A single herald patch (oval-shaped, rose-colored patches with slight scale) on the trunk, followed by smaller similar lesions [5].
Gianotti-Crosti syndrome	Lichenoid, flesh-colored to red papules on the face, buttocks, and extremities, coalescing to form plaques [8].
Erythema infectiosum	"Slapped cheek" rash, followed by pink papules and macules in a lacy, reticular pattern on the extremities [5,8].
Rocky Mountain spotted fever	Pale red or rose-colored maculopapular rash starting on the wrists and ankles and progressing to petechia [8].
Scarlet fever	Erythema with sandpaper-like papules after the fever and sore throat, starting on the upper trunk [5].
Staphylococcal scalded skin syndrome	Generalized macular erythema without mucosal involvement, with perioral and intertriginous erythema becoming vesicular and bullous, rapidly exfoliating within 2 to 3 days [8].
Tinea corporis	Erythematous annular patch or plaque with a raised, scaly border and central clearing [5].
Kawasaki disease	Generalized erythema with fever, conjunctivitis, changes in the oropharyngeal mucosa and peripheral extremities, and cervical lymphadenopathy [8].
Drug-induced exanthema	Maculopapular exanthem after 7-12 days post exposure (common culprits: beta-lactams and non-steroidal anti-inflammatory drugs) [9].
Erythema multiforme	Target lesions [8].

While the typical form of scarlet fever is usually easy to identify, diagnosing atypical cases can be challenging because they resemble other conditions. Fernández Romero et al. reported that 10% of scarlet fever cases presented with a rash only [10]. In this retrospective study, 20 out of 91 scarlet fever patients presented with atypical exanthema, but erythema limited to the distal extremities, as seen in this case, was not included, suggesting that this case represents a rare manifestation. Therefore, even when a rash is the sole symptom, considering scarlet fever in the differential diagnosis is crucial for early diagnosis and treatment to prevent both suppurative complications (e.g., otitis media, peritonsillar abscess, pneumonia, endocarditis, and meningitis) and non-suppurative complications (e.g., rheumatic fever and streptococcal glomerulonephritis) [2,6]. In this case, other conditions presenting with a rash were initially considered, but the patient’s history of exposure and pharyngeal erythema, which was painless and therefore unnoticed even by the patient, provided key diagnostic clues.

## Conclusions

It is essential to diagnose scarlet fever early to prevent both suppurative and non-suppurative complications. The classical features of scarlet fever are often incomplete, and patients may present with skin manifestations as the primary symptom. Clinicians should remain vigilant when evaluating erythema, considering not only the skin findings but also the patient's history and a thorough physical examination. Additionally, awareness of local epidemiological data and potential outbreaks is essential for prompt diagnosis and appropriate management.
